# Total Flavonoids from *Clinopodium chinense* (Benth.) O. Ktze Protect against Doxorubicin-Induced Cardiotoxicity *In Vitro* and *In Vivo*

**DOI:** 10.1155/2015/472565

**Published:** 2015-02-16

**Authors:** Rong Chang Chen, Xu Dong Xu, Xue Zhi Liu, Gui Bo Sun, Yin Di Zhu, Xi Dong, Jian Wang, Hai Jing Zhang, Qiang Zhang, Xiao Bo Sun

**Affiliations:** ^1^Institute of Medicinal Plant Development, Chinese Academy of Medical Science, Peking Union Medical College, No. 151, North Road Malianwa, Haidian District, Beijing 100094, China; ^2^Academy of Forestry, Baishan, Jilin 134302, China; ^3^Academy of Chinese Materia Medica, Wenzhou Medical College, Wenzhou, Zhejiang 325035, China; ^4^Harbin University of Commerce, Xuehai Street, Songbei District, Harbin, Heilongjiang 150028, China; ^5^Department of Pharmacology, Heilongjiang University of Chinese Medicine, Harbin, Heilongjiang 150040, China

## Abstract

Doxorubicin has cardiotoxic effects that limit its clinical benefit in cancer patients. This study aims to investigate the protective effects of the total flavonoids from *Clinopodium chinense* (Benth.) O. Ktze (TFCC) against doxorubicin- (DOX-) induced cardiotoxicity. Male rats were intraperitoneally injected with a single dose of DOX (3 mg/kg) every 2 days for three injections. Heart samples were collected 2 weeks after the last DOX dose and then analyzed. DOX delayed body and heart growth and caused cardiac tissue injury, oxidative stress, apoptotic damage, mitochondrial dysfunction, and Bcl-2 expression disturbance. Similar experiments in H9C2 cardiomyocytes showed that doxorubicin reduced cell viability, increased ROS generation and DNA fragmentation, disrupted mitochondrial membrane potential, and induced apoptotic cell death. However, TFCC pretreatment suppressed all of these adverse effects of doxorubicin. Signal transduction studies indicated that TFCC suppressed DOX-induced overexpression of p53 and phosphorylation of JNK, p38, and ERK. Studies with LY294002 (a PI3K/AKT inhibitor) demonstrated that the mechanism of TFCC-induced cardioprotection also involves activation of PI3K/AKT. These findings indicated the potential clinical application of TFCC in preventing DOX-induced cardiac oxidative stress.

## 1. Introduction

Doxorubicin (DOX) is an anthracycline derivative widely used to treat various cancers, including acute leukemias, malignant lymphomas, and a variety of solid tumors [1, 2]. However, the prolonged use of DOX may cause cardiotoxicity and lead to cardiomyopathy and heart failure, which severely limit its clinical applications [3]. Numerous mechanisms have been proposed and oxidative stress is believed to play a key role in the intracellular signaling pathways and apoptosis in this pathophysiology [4–6]. Antioxidants reportedly exert protective effects on DOX-treated cardiomyocytes [7]. However, no single active ingredient has been clinically proved effective and safe for preventing this severe side effect. Therefore, the discovery of plant extracts with cardioprotective effects is important to continue the clinical use of DOX.

Intrinsic pathway and extrinsic pathway are the two canonical apoptosis signaling pathways. The activation of p53-initiated intrinsic pathway has been implicated in DOX-induced cardiotoxicity and subsequent chronic cardiomyopathy [8]. It is also known that oxidative stress induced activation of mitogen-activated protein kinase (MAPK) signaling pathways which are the primary intermediates for the induction of apoptosis [9]. MAPK includes three major signaling cascades: the extracellular signal-related kinases (ERK1/2), the c-Jun N-terminal kinases (JNK), and the p38 kinase (p38). The literature suggests that DOX induces the activation of MAPKs in cardiac pathophysiology [10]. In the present study, we investigated the function of MAPKs during DOX-induced myocardium apoptosis. The phosphoinositide 3-kinase (PI3K)/AKT signaling pathway is an important signaling transduction pathway that plays an important role in the development heart disease and protects cells undergoing apoptosis [11, 12]. We also studied the effect of DOX on this signaling pathway in the present study.

*Clinopodium chinense *(Benth.) O. Ktze is a traditional Chinese herbal medicine and is originally documented in the “Jiuhuang Herbal,” which belongs to the family Labiatae. It was reported that the stem and leave of* Clinopodium chinense *(Benth.) O. Ktze possess various pharmacological effects, such as anti-inflammatory and immunity [13, 14], lowering blood glucose [15], and antitumor and antiradiation [16, 17]. The major components have been identified in the* C. chinense* (Benth.) O. Ktze, including flavonoids, triterpenoid saponins, and volatile oil [16, 18]. Total flavonoids from* C. chinense* (Benth.) O. Ktze (TFCC) play an important role for the treatment of cardiovascular disease [16]. The instant hypotensive effect of TFCC has been found in normal rabbit. TFCC perfusion reduced contractility and slow rhythm of the heart isolated from toad. Besides, TFCC could also suppress elevation of ECG ST-T induced by pituitrin and improve the ability of hypoxia tolerance in the heart of rabbit injected with epinephrine and norepinephrine [19]. The alcohol extract from* C. chinense* (Benth.) O. Ktze can effectively reduce oxidative stress injury in HUVECs induced by H_2_O_2_ [20]. However, there has been no study about the protective effect of* C. chinense *(Benth.) O. Ktze on DOX-induced cardiotoxicity. The present study aimed to examine the protective effects of TFCC against DOX-induced cardiotoxicity* in vitro* and* in vivo* and determine its underlying mechanisms.

## 2. Materials and Methods

### 2.1. Ethics Statement

All animal experiments were approved by the Medical Ethics Committee of Peking Union Medical College and were in accordance with the National Institutes of Health Regulations for the Care and Use of Animals. All efforts were made to minimize suffering.

### 2.2. Plant Materials

*C. chinense *(Benth.) O. Ktze was purchased from Chinese Medicinal Material Markets (Bozhou, China) in 2012. Effective plant overground part identification was provided by Professor Shichun Yu of the School of Pharmacy, Anhui University of Chinese Medicine. The voucher specimen (20130312) was deposited in the Key Laboratory of Bioactive Substances and Resources Utilization of Chinese Herbal Medicine, Ministry of Education, Beijing, China.

The dried overground part (1 kg) of* C. chinense* was crushed with a grinder (Tianjin Taisite Instrument Co., Ltd., Tianjin, China) and extracted twice with 8 L of 70% ethanol (v/v) at 70°C for 2 h. The extract was filtered and evaporated to remove ethanol completely. The residue was suspended in water and successively extracted with petroleum ether and ethyl acetate. Approximately 56 g residue of ethyl acetate was loaded on a polyamide column (700 mm × 75 mm) and then eluted with 2 L of 20% ethanol, 8 L of 20% ethanol (v/v), 15 L of 50% ethanol (v/v), and 15 L of 70% ethanol (v/v). The effluents of 50% and 70% ethanol were collected and evaporated at 55°C under vacuum. At last, we obtained 26 g of the 40% and 70% ethanol fractions of the total flavones.

### 2.3. Ultraperformance Liquid Chromatography (UPLC) Analysis

UPLC was performed using an Acquity UPLC system (Waters, USA) with an Agilent phenyl column (100 mm × 2.1 mm i.d., 1.7 *μ*m). The mobile phase was composed of water with 0.2% formic acid (A) and acetonitrile (B) with the following gradient elutions: 0 min to 3 min, 2% to 16% B; 3 min to 6 min, 16% to 20% B; 6 min to 12 min, 20% B; 12 min to 13 min, 20% to 26% B; 13 min to 16 min, 26% to 35% B; 16 min to 20 min, 35% to 50% B; and 20 min to 23 min, 50% to 100% B. The flow rate was set at 0.4 mL/min, the column temperature was 40°C, and the sample injection volume was 10 *μ*L.

### 2.4. Mass Spectrometry

Mass spectrometry detection was performed on a Synapt G2 MS system (Waters, USA) equipped with an electrospray ionization (ESI) source. Masslynx 4.1 (Waters, USA) was used to control the instrument. Nitrogen gas was used for nebulization. The detection mode of the flight tube was selected to be a “V” pattern. The negative ion spectra of the column elute were recorded within the range of 100 *m*/*z* to 1200 *m*/*z*. The ESI source conditions were as follows: capillary voltage, 2.5 kV; sampling cone voltage, 40 V; extraction cone voltage, 4.0 V; ESI source temperature, 120°C; desolvation temperature, 450°C; cone gas flow, 50 L/h; and desolvation gas flow, 800 L/h. The lock mass compound used was leucine enkephalin (554.2615 *m*/*z*), and the interval scan time was 0.02 s.

### 2.5. Drugs and Reagents

DOX was purchased from Shenzhen Main Luck Pharmaceuticals, Inc. (Shenzhen, China). Cell culture materials were purchased from GIBCO (Grand Island, NY). Caspase-3 fluorometric assay kits were acquired from BioVision (CA, USA). The fluorescent dye (JC-1) was purchased from Molecular Probes (Eugene, OR). The kits for determining malondialdehyde (MDA), glutathione peroxidase (GSH-Px), superoxide dismutase (SOD), and catalase (CAT) were purchased from Nanjing Jiancheng Institute of Biological Engineering (Nanjing, China). The kits for measuring lactate dehydrogenase (LDH), creatine kinase (CK), and aspartate aminotransferase (AST) were obtained from Biosino Biotechnology and Science Inc. (Hong Kong, China). All antibodies were purchased from Santa Cruz Biotechnology (Santa Cruz, CA), and other chemicals were purchased from Sigma (St. Louis, MO).

### 2.6. Animals and Pharmacological Treatment* In Vivo*

Male Sprague-Dawley (SD) rats weighing 220 g to 250 g were used in the experiments. All SD rats were randomly assigned to the following groups: control (distilled water), TFCC (80 mg/kg), DOX, and TFCC + DOX (20, 40, and 80 mg/kg). These rats were intragastrically treated with distilled water or TFCC for 15 d and then intraperitoneally injected with normal saline or a single dose of DOX (3 mg/kg) every two days for a total of three injections. The TFCC group was only treated with the highest TFCC concentration (80 mg/kg). After 14 d of the first administration of DOX, the rats were euthanized for morphological and cellular studies. The body and heart weights were measured. Serum samples were assayed for the determination of serum cardiac enzymes (e.g., CK, AST, and LDH) by kits using an automatic chemical analyzer (7060, Hitachi, Tokyo, Japan). The left ventricle was excised for histopathological examination, and myocardial homogenates were prepared for MDA, GSH-Px, CAT, and SOD detection by the corresponding kits.

### 2.7. Heart Histopathological Examination

The heart apex was trimmed and embedded in paraffin blocks after fixation with 4% paraformaldehyde. The heart apex was sectioned and stained with hematoxylin and eosin. The structure was then examined under a light microscope (CKX41, 170 Olympus, Tokyo, Japan) by a pathologist blinded to the groups under study.

### 2.8. Cell Culture and Treatment

Rat embryonic cardiomyoblast-derived H9c2 cells were cultured in DMEM supplemented with 10% (v/v) fetal bovine serum, 2 mM L-glutamine, 100 U/mL penicillin, and 100 mg/mL streptomycin. The cells were cultured in a humidified incubator with 95% air/5% CO_2_ at 37°C [21]. For all experiments, the cells were plated at an appropriate density according to the experimental design and grown for 36 h before treatment. After being treated with TFCC (0 *μ*g/mL to 50 *μ*g/mL) with or without 1 *μ*M DOX (4 h after) for 24 h, the cells were harvested at 4°C for further molecular and biochemical analyses.

### 2.9. Cell Viability Analysis

Cell viability was determined by MTT assay [22]. Briefly, the cells were seeded in 96-well plates at 1 × 10^5^ cells/well. After being pretreated with TFCC at different concentrations for 4 h and incubated with 1 *μ*M DOX for 24 h, the cells were incubated with 20 *μ*L 5 mg/mL MTT solution at 37°C for 4 h. The supernatants were discarded, and the metabolized MTT in each well was dissolved in 150 *μ*L of DMSO. Optical density was measured at 570 nm on a microplate reader (BioTek, Vermont).

### 2.10. Measurement of LDH and MDA Levels and SOD, CAT, and GSH-Px Activities

H9c2 cells were cultured in six-well plates at 3 × 10^5^ cells/well. After the experimental procedures, the supernatant was used to measure the level of LDH release using an LDH assay kit. The cells were collected, ultrasonicated, and centrifuged at 1000 rpm for 5 min at 4°C. The supernatant was used to assess activities of MDA, SOD, CAT, and GSH-Px according to the corresponding detection kits (Nanjing Jiancheng Bioengineering Institute, Nanjing, China).

### 2.11. Hoechst 33342 Staining

Hoechst 33342 staining, which distinguishes apoptotic cells from normal cells on the basis of nuclear chromatin condensation and fragmentation, was used to qualitatively analyze the apoptotic cells. H9c2 cells were cultured on cover slips in 24-well plates for 36 h. After treatment, the cells were incubated with 5 mg/mL Hoechst 33342 at 37°C for 15 min, washed twice with phosphate-buffered saline (PBS), and then observed via fluorescence microscopy (Leica, Heidelberg, Germany).

### 2.12. TUNEL Staining

Cell apoptosis in the heart tissue and H9c2 cells was detected using TUNEL assay according to the manufacturer's protocol. After dewaxing and rehydration, the heart sections were incubated with proteinase K (20 mg/mL) at room temperature for 15 min. After rinsing with PBS, the slices were incubated with working-strength terminal deoxynucleotidyl transferase enzyme at 37°C for 1 h in a humidified chamber. After rinsing in a stop/wash buffer, the sections were incubated with working-strength antidigoxigenin conjugate at room temperature for 30 min. The slices were stained with 4′6-diamidino-2-phenylindole and then observed under a fluorescence microscope (Leica, Heidelberg, Germany). H9c2 cells were cultured on cover slips for 36 h. After the treatment, the cells were fixed in 1% paraformaldehyde at room temperature for 30 min. Subsequently, the cells were treated with a permeabilizing solution (0.1% 162 Triton X-100) at 4°C for 2 min, incubated in the TUNEL reaction mixture at 37°C for 60 min, and then visualized by fluorescence microscopy (Leica, Heidelberg, Germany).

### 2.13. Measurement of Mitochondrial Membrane Potential (ΔΨ_*m*_)

The change of mitochondrial transmembrane potential was determined using JC-1. JC-1 accumulates to form J-aggregates and emits red fluorescence when there are mitochondria with high membrane potential. However, JC-1 emits green fluorescence when this compound dissociates into monomers in mitochondria with no cross-membrane electrochemical gradient. Therefore, the ratio of green to red fluorescence can be a reliable estimate of mitochondrial ΔΨ_*m*_ impairment. H9c2 cells from different treatment groups were washed with PBS and incubated with JC-1 (1 *μ*M) in DMEM at 37°C for 20 min. After washing with PBS, the cells were immediately analyzed under a fluorescence microscope. The ratio of aggregated and monomeric JC-1 was used to quantify the change of mitochondrial membrane potential. Decreased JC-1 ratio represents depolarization of the mitochondria, indicating a decrease in mitochondrial membrane potential.

### 2.14. Caspase-3 Activity Assessments

Caspase-3 activation was measured using a fluorescein active caspase-3 staining kit according to the manufacturer's instructions (BioVision, CA, USA). After treatment, approximately 300 *μ*L (1 × 10^6^ cells/mL) of the cultures was incubated with 1 *μ*L of the substrate FITC-DEVD-FMK at 37°C for 1 h. These cultures were then centrifuged at 3000 rpm for 5 min, and the supernatant was removed. After washing twice with cold PBS, the cells were resuspended in 100 *μ*L of wash buffer. The cell suspension was transferred to each well in a black microtiter plate. Fluorescence intensity was detected using a microplate reader at excitation and emission wavelengths of 485 and 535 nm, respectively.

### 2.15. Detection of Intracellular ROS Production

The total ROS detection kit was used to monitor the level of intracellular ROS according to the manufacturer's protocol (Enzo Life Sciences, Farmingdale, NY). After treatment, the cells were harvested, placed in 10 mL round bottom polystyrene tubes, and then washed with 1x wash buffer. The cells were centrifuged at 1000 g for 5 min at room temperature, and the supernatant was discarded. The cells were resuspended in 500 mL of ROS detection solution, stained at 37°C for 30 min in the dark, and then analyzed by flow cytometry.

### 2.16. Western Blot Analysis

Heart tissues or H9c2 cells were lysed on ice with tissue or cell protein extraction reagent that contains a 0.1 mM dithiothreitol and proteinase inhibitor cocktail. The protein concentration was determined using a BCA kit (Pierce Corporation, Rockford, USA). Equal amounts of protein fractions were separated by 12% SDS-PAGE and then transferred onto nitrocellulose membranes (Millipore Corporation, USA) in Tris-glycine buffer at 100 V for 55 min. The membranes were blocked with 5% (w/v) nonfat milk powder in Tris-buffer that contains 0.05% (v/v) Tween-20 (TBST) at room temperature for 2 h. After incubation overnight with appropriate primary antibodies at 4°C, the membrane was washed thrice with TBST, incubated with secondary antibodies for 2 h at room temperature, and then washed again thrice with TBST. Protein blots were developed using an enhanced chemiluminescence solution. The protein expression levels were visualized with Image Lab Software (Bio-Rad, USA).

### 2.17. Statistical Analysis

Results from at least three independent experiments were expressed as mean ± SE. Statistical comparisons between different groups were measured by using Student's *t*-test or ANOVA with Prism 5.00 software. Statistical significance was considered at *P* < 0.05.

## 3. Results

### 3.1. Characterization of TFCC Using UPLC/Q-TOF-MS/MS

**Figure 1:**
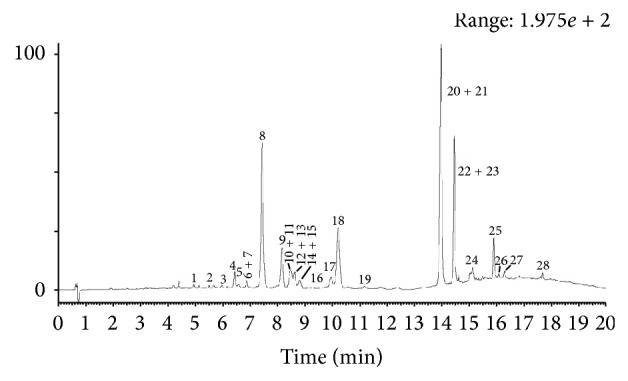
Characterization of total flavonoids from* Clinopodium chinense* (Benth.) O. Ktze (TFCC) using UPLC/Q-TOF-MS/MS.

TFCC were characterized using UPLC/Q-TOF-MS/MS, in which 28 peaks were observed (Figure 1). The 28 peaks were assigned by MS fragments and the previous literatures [23, 24] (Table 1).

### 3.2. TFCC Reduced DOX-Induced Myocardial Injury in Rats

**Figure 2:**
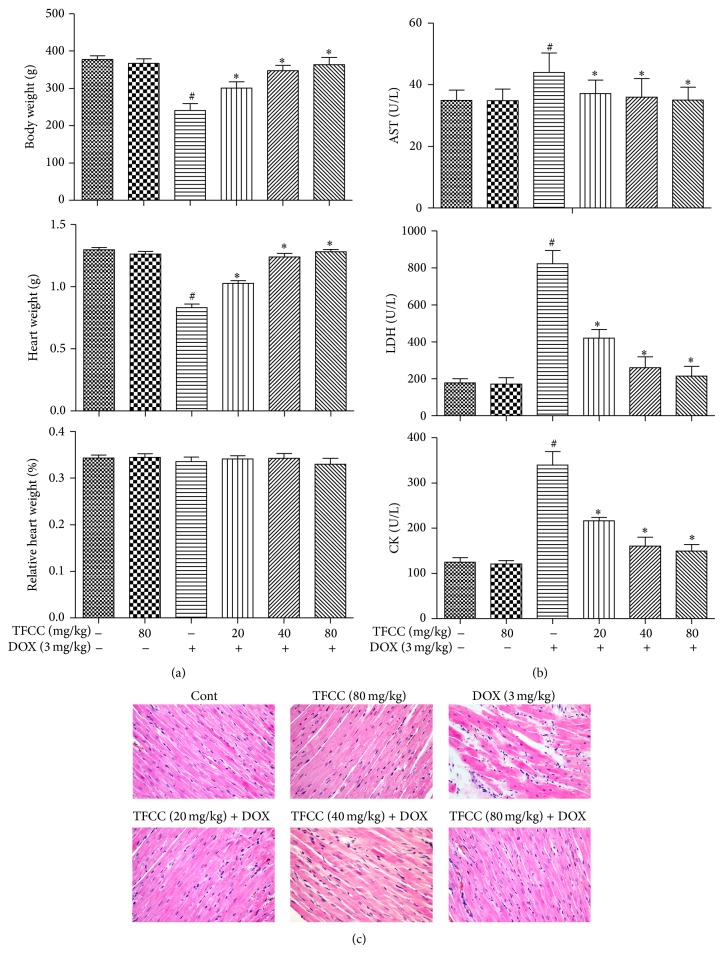
Effects of TFCC on DOX-induced myocardial injury* in vivo*. Rats were treated with vehicle or DOX (i.p., 3 mg/kg every other day) with or without TFCC pretreatment (20, 40, and 80 mg/kg i.p.). At day 14, body weight, absolute heart weight, and relative heart weight index (heart weight-to-body weight ratio) were determined (a); effects of TFCC and DOX on AST, LDH, and CK activities were measured (b); effects of TFCC and DOX on histological changes in rat hearts by HE staining (200x) (c). Results are represented as the mean ± SE. ^#^ *P* < 0.05 relative to the control group, and ^*^ *P* < 0.05 relative to the DOX group.

We first measured the general toxicities of DOX and TFCC* in vitro*. As shown in Figure 2(a), the rats treated with DOX showed significantly less body and heart weights after 14 d. By contrast, TFCC pretreatment (20, 40, and 80 mg/kg i.p.) resulted in a dose-dependent recovery of body and heart weights. However, no significant difference in the relative heart weight index (heart weight-to-body weight ratio) was detected among all six groups after 14 d (Figure 2(a)).

The levels of cardiac enzymes (CK, AST, and LDH) in serum were evaluated to assay the cardioprotective effects of TFCC. TFCC significantly ameliorated the DOX-induced increase in serum cardiac enzyme levels in a dose-dependent manner. TFCC treatment alone did not cause any apparent abnormalities (Figure 2(b)). Based on the histological assessments of the different cardiac segments of the experimental animals, we observed that DOX injection disorganized the normal radiating pattern of cell plates in the heart. TFCC reduced such changes, and the hearts appeared similar to those of the normal group.

### 3.3. TFCC Protected against DOX-Induced Cytotoxicity in H9c2 Cells

**Figure 3:**
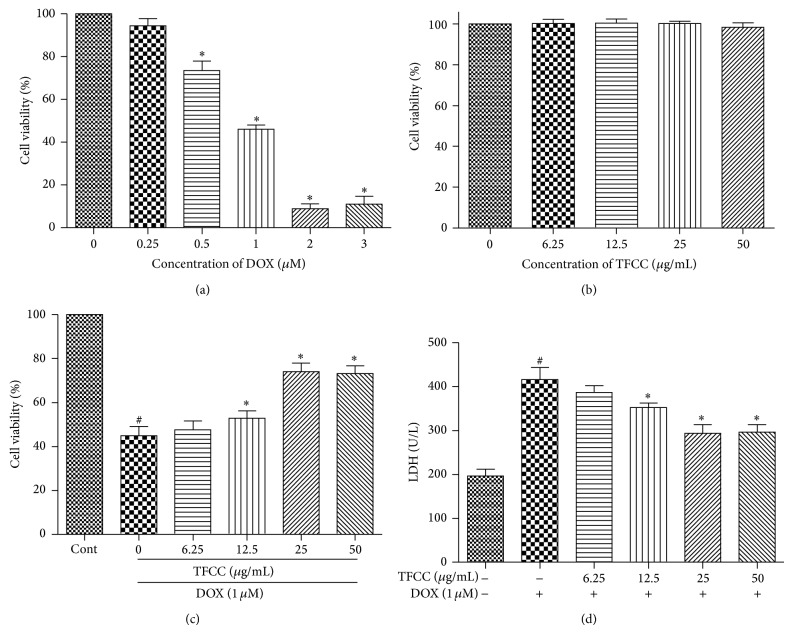
Effects of DOX and TFCC on cardiomyocyte viability and LDH release* in vitro*. H9c2 cells were treated for 24 h with different concentrations of DOX, and cell viability was measured by MTT assay (a). H9c2 cells were treated with different concentrations of TFCC for 4 h, and cell viability was measured by MTT assay (b). Control and TFCC-treated cells were further exposed to 1 *μ*M DOX for 24 h. Cell viability (c) and LDH release (d) were measured. Results are represented as the mean ± SE. ^#^ *P* < 0.05 relative to the control group, and ^*^ *P* < 0.05 relative to the DOX group.

We evaluated the effect of DOX on cell viability using MTT assay. H9c2 cells were treated with different concentrations of DOX (0.25, 0.5, 1, 2, and 3 *μ*M) for 24 h. As shown in Figure 3(a), cell viability significantly decreased at 0.5 *μ*M DOX and decreased by approximately 50% at 1 *μ*M DOX. DOX caused severe cell damage, and cell viability was only 11.1% and 9.1% at 2 and 3 *μ*M, respectively (Figure 3(a)). Therefore, 1 *μ*M DOX for 24 h was used for subsequent experiments. Before testing the protective effects of TFCC against DOX-induced cytotoxicity in H9c2 cells, we analyzed the direct effect of TFCC on H9c2 cells. Cells were treated with different concentrations of TFCC (6.25, 12.5, 25, and 50 *μ*g/mL) for 4 h. As shown in Figure 3(b), no significant difference in cell viability was observed between the TFCC-treated and control groups. Therefore, the tested TFCC concentrations could not induce cell injury in H9c2 cells and will be used in the next experiments. As shown in Figures 3(c) and 3(d), DOX significantly reduced cell viability and increased LDH release, whereas pretreatment with 25 *μ*g/mL TFCC significantly maintained cell viability at approximately 78% and reduced LDH release. A high concentration of TFCC (50 *μ*g/mL) showed no additional benefit. Therefore, a 25 *μ*g/mL dose was selected for subsequent experiments* in vitro*.

### 3.4. TFCC Enhanced Antioxidant Capacity in H9c2 Cells and Cardiac Tissues

**Figure 4:**
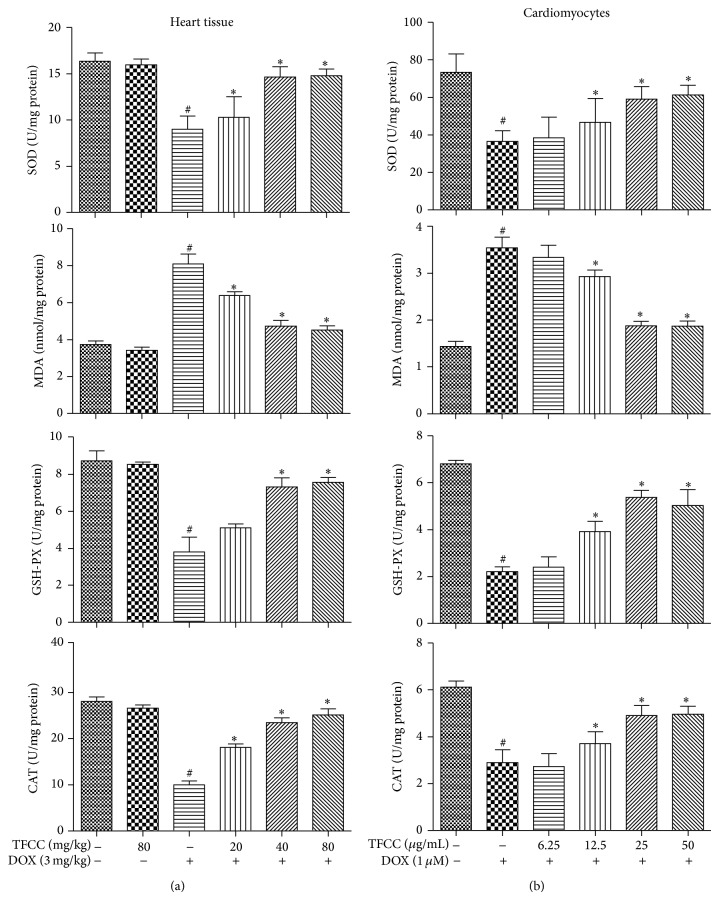
Effects of DOX and TFCC on oxidative stress damage* in vivo* and* in vitro*. Effects of TFCC and DOX on SOD, MDA, CAT, and GSH-Px activities in rat heart tissues (a) and H9c2 cells (b) were measured by corresponding detection kits. Results are represented as the mean ± SE. ^#^ *P* < 0.05 relative to the control group, and ^*^ *P* < 0.05 relative to the DOX group.

The effects of DOX and TFCC with respect to oxidative stress damage were further evaluated* in vivo* and* in vitro*. DOX induced oxidative stress damage in both rat heart and H9c2 cells, as indicated by decrease in SOD, CAT, and GSH-Px activities and increase in MDA production (Figures 4(a) and 4(b)). These actions were dose-dependently ameliorated by TFCC. These findings proved the efficacy of TFCC in alleviating DOX-induced oxidative stress.

### 3.5. TFCC Reduced DOX-Induced ROS Generation in H9c2 Cells

**Figure 5:**
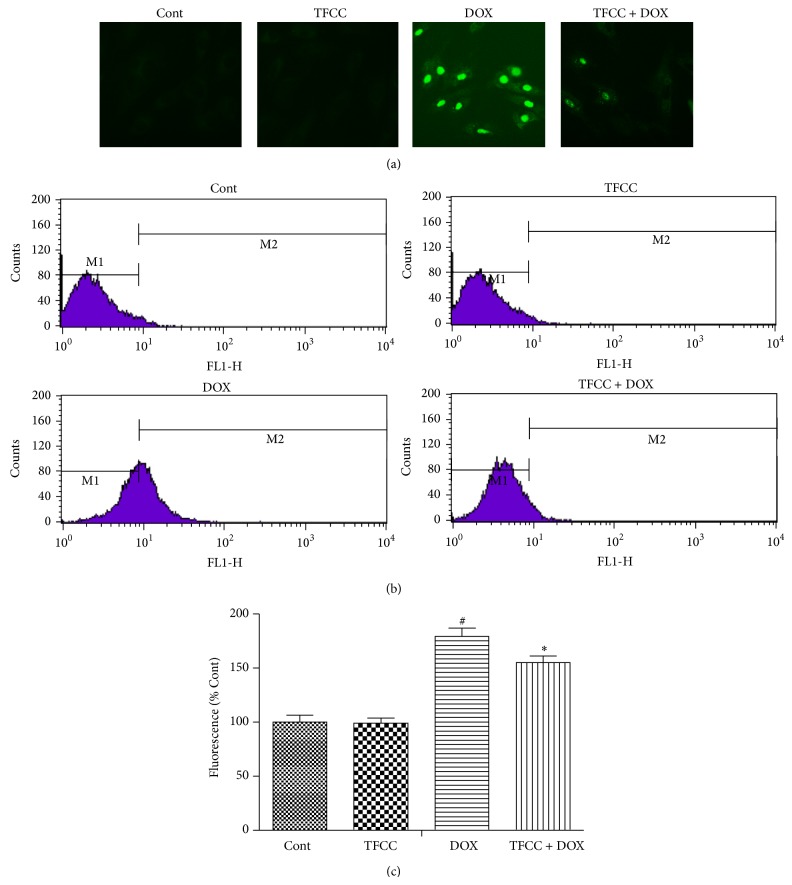
Effects of DOX and TFCC on intracellular ROS generation. After 4 h of pretreatment with or without TFCC (25 *μ*g/mL), the cells were exposed to DOX for 24 h and were assayed using ROS detection kit (a). Representative ROS staining histogram of H9c2 cells using flow cytometric analysis (b). Statistical analysis of ROS generation (c). The results are represented as the mean ± SE. ^#^ *P* < 0.05 relative to the control group, and ^*^ *P* < 0.05 relative to the DOX group.

Flavonoids can change the cellular ROS level [25, 26]. Therefore, we determined whether or not TFCC show a potent ROS-scavenging effect. The results of fluorescence assay (Figure 5(a)) and quantitative flow cytometry (Figures 5(b) and 5(c)) showed that H9c2 cells exposed to DOX displayed a significant increase in ROS production (approximately 76% versus control). By contrast, TFCC pretreatment effectively reduced DOX-induced ROS production. However, TFCC alone had no effect on ROS levels compared with the control treatment.

### 3.6. TFCC Protected against DOX-Induced Apoptosis* In Vivo* and* In Vitro*

**Figure 6:**
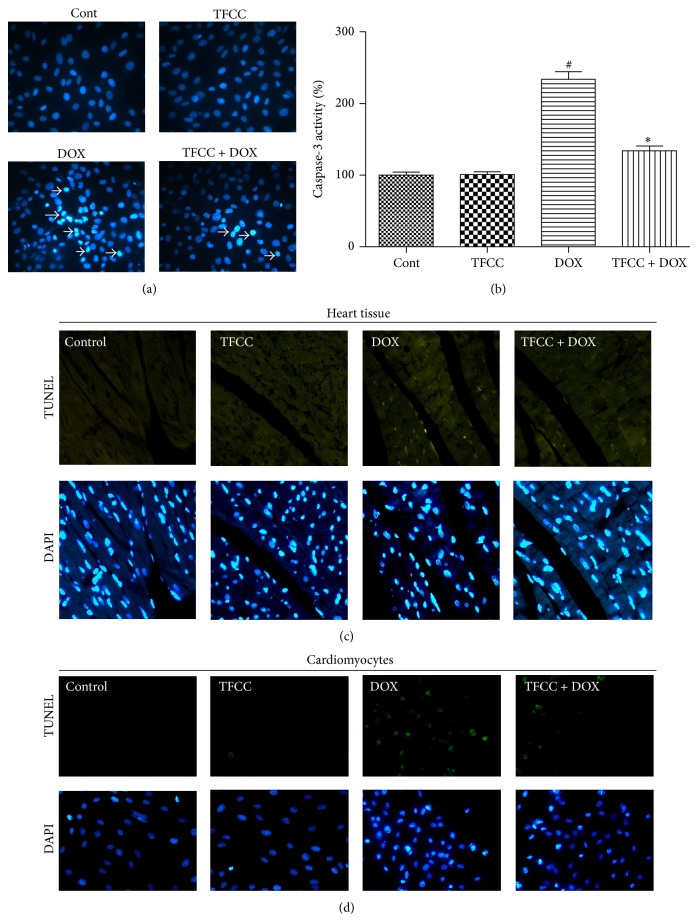
Effects of DOX and TFCC on apoptosis* in vivo* and* in vitro*. H9c2 cells were treated with vehicle or DOX (1 *μ*M) with or without TFCC (25 *μ*g/mL for 4 h prior to DOX exposure). After 24 h, Hoechst 33342 staining (a) and TUNEL assay (d) were used to measure the apoptosis in H9c2 cells. Caspase-3 activity (units per microgram of protein) was assayed as described in Materials and Methods (b). Rats were treated with vehicle or DOX (i.p., 3 mg/kg every other day) with or without TFCC pretreatment (40 mg/kg i.p. before DOX administration). At day 14, heart tissue section was obtained, and TUNEL assay of heart tissue was measured (c). Cont, vehicle treatment; TFCC, TFCC treatment; DOX, doxorubicin treatment; TFCC + DOX, TFCC and DOX cotreatment. The results are represented as the mean ± SE. ^#^ *P* < 0.05 relative to the control group, and ^*^ *P* < 0.05 relative to the DOX group.

We examined the nuclear morphology after Hoechst 33342 staining to determine the effect of TFCC on DOX-induced apoptosis in H9c2 cells. DOX induced nuclear changes in H9c2 cells with heterogeneous intensity and chromatin condensation. Pretreatment with 25 *μ*g/mL TFCC for 4 h exhibited a strong antiapoptotic effect (Figure 6(a)). Caspase-3 activity was significantly upregulated in the cells treated with DOX and was inhibited by TFCC pretreatment (Figure 6(b)). Afterward, we examined the DNA fragmentation pattern to show the apoptotic changes in H9c2 cells and rat myocardial tissues. DOX caused DNA ladder fragmentation (Figure 6(c)), whereas TFCC treatment effectively reduced DNA laddering in the DOX-treated cardiomyocytes. However, TFCC alone showed no significant effect on apoptotic processes in both* in vivo* and* in vitro* experiments.

### 3.7. TFCC Prevented DOX-Induced Apoptosis through Mitochondrion-Dependent Apoptotic Pathway

**Figure 7:**
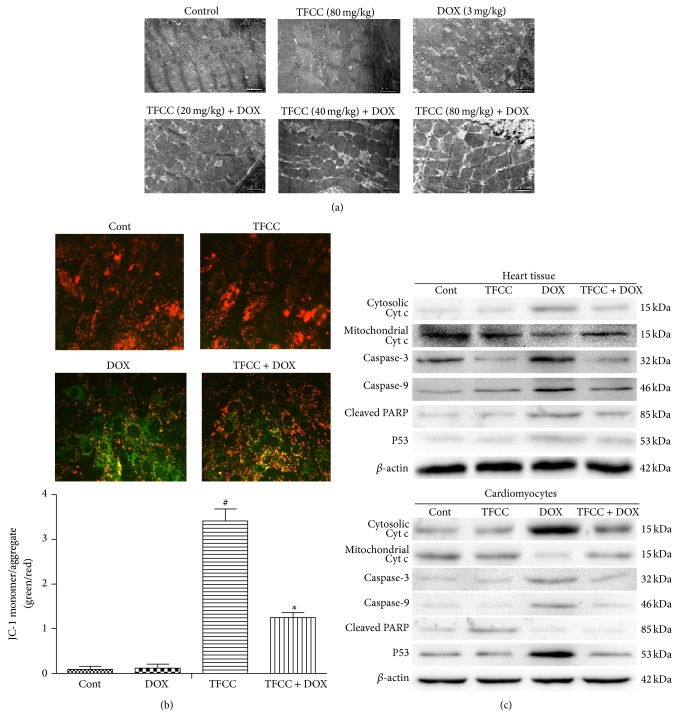
Effects of DOX and TFCC on mitochondrial apoptotic pathway* in vivo* and* in vitro*. Rats were treated with vehicle or DOX (i.p., 3 mg/kg every other day) with or without TFCC pretreatment (20, 40, and 80 mg/kg i.p.). At day 14, the ultrastructure of cardiomyocytes was observed using electron microscope (a). H9c2 cells were treated with vehicle or DOX (1 *μ*M) with or without TFCC (25 *μ*g/mL for 4 h prior to DOX exposure). After 24 h, cells were stained with JC-1 dye and were visualized by fluorescence microscopy. Quantitative analysis of JC-1 staining was evaluated (green to red fluorescence ratio) (b). Cytochrome c (cyt c) in mitochondrial and cytosolic protein extracts and caspase-3/9 and PARP levels in rat heart tissues and H9c2 cells were determined using Western blot analysis (c). Cont, vehicle treatment; TFCC, TFCC treatment; DOX, DOX treatment; TFCC + DOX, TFCC and DOX cotreatment. The results are represented as the mean ± SE. ^#^ *P* < 0.05 relative to the control group, and ^*^ *P* < 0.05 relative to the DOX group.

The ultrastructural changes in cardiomyocytes were examined to evaluate the myocardial damage induced by chronic DOX treatment. The control and TFCC groups showed no significant morphological abnormalities. However, DOX treatment resulted in myofibril loss, cytoplasm vacuolization, and mitochondrial swelling with membrane disruption and these results were significantly attenuated by TFCC pretreatment (Figure 7(a)). Mitochondrion is the key organelle related to cell energy supply and cellular apoptosis. A decrease in the mitochondrial membrane potential causes membrane depolarization and triggers a cascade of apoptotic signaling [27]. In the present study, JC-1 staining showed that TFCC pretreatment abated DOX-induced decrease in ΔΨ_*m*_ in H9c2 cells (Figure 7(b)). Disruption of mitochondrial membrane permeability results in mitochondrial cytochrome c release to the cytosol, which in turn activates the apoptotic effector caspase-3 to induce cell apoptosis [28]. Immunofluorescence results showed that DOX caused mitochondrial cytochrome c release into the cytosol and subsequent activation of caspase-9/3 and poly ADP-ribose polymerase (PARP) cleavage. In contrast, TFCC inhibited DOX-induced mitochondrial cytochrome c release and prevented the cleavage of caspase-9/3 and PARP (Figure 7(c)).

### 3.8. TFCC Preserved DOX-Induced Disturbance in the Expression Patterns of Bcl-2 and Bax* In Vivo* and* In Vitro*

**Figure 8:**
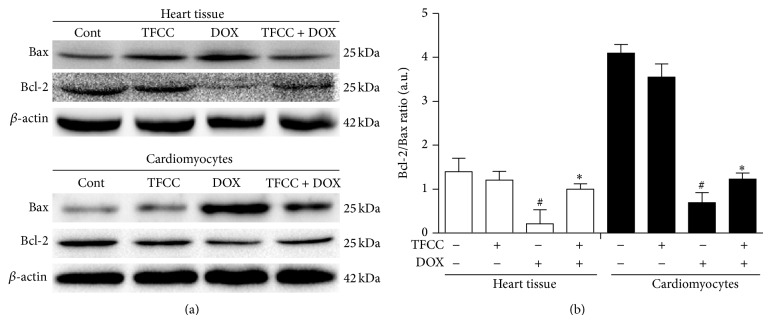
Effects of DOX and TFCC on Bcl-2 and Bax expression patterns* in vivo* and* in vitro*. After treatment, Bcl-2 and Bax protein levels in rat heart tissues and H9c2 cells were determined using Western blot analysis (a). Quantitative analysis of the ratio of Bcl-2 to Bax in protein expression (b). Cont, vehicle treatment; TFCC, TFCC treatment; DOX, DOX treatment; TFCC + DOX, TFCC and DOX cotreatment. The results are represented as the mean ± SE. ^#^ *P* < 0.05 relative to the control group, and ^*^ *P* < 0.05 relative to the DOX group.

TFCC showed protective effect against DOX-induced mitochondrial damage. Therefore, we subsequently evaluated the expression levels of Bcl-2 proteins, mainly including the proapoptotic Bax and the antiapoptotic Bcl-2, which are upstream regulators of mitochondrial potential [29]. The results of Western blot assay showed that DOX caused elevated Bax protein levels and concomitance with decreased Bcl-2 protein levels in both rat heart tissues and H9c2 cells (Figure 8(a)). However, TFCC mitigated DOX-induced Bax upregulation and Bcl-2 downregulation. Statistical calculation of the Bcl-2 to Bax ratio with respect to protein levels revealed that TFCC alleviated the DOX-induced disturbance in Bcl-2 and Bax protein expression in both* in vivo* and* in vitro* experiments (Figure 8(b)). However, TFCC alone exerted no significant effects on both Bax and Bcl-2 protein levels.

### 3.9. Effects of TFCC and DOX on Phosphorylation of Different MAPKs* In Vitro*

**Figure 9:**
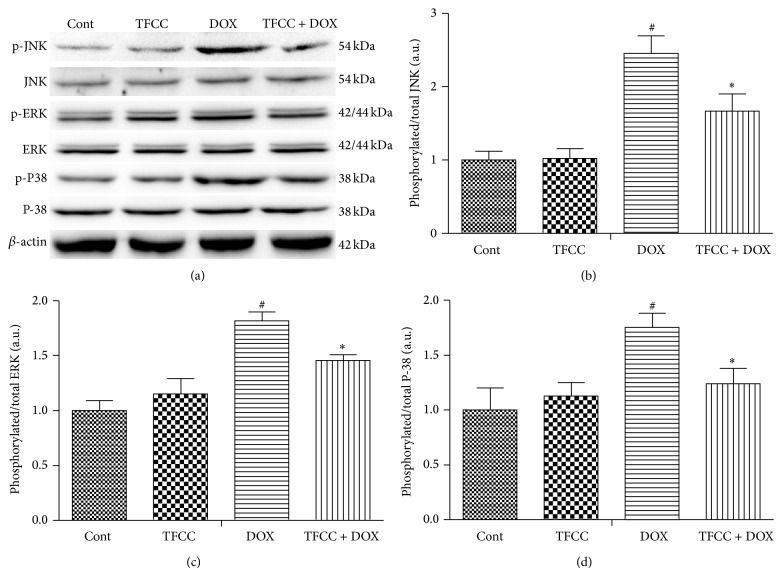
The effects of DOX and TFCC on phosphorylation of different MAPKs* in vitro*. H9c2 cells were treated with vehicle or DOX (1 *μ*M) with or without TFCC (25 *μ*g/mL for 4 h prior to DOX exposure) for 24 h. The protein levels of phosphorylated and total ERK1/2, p38, and JNK were measured using Western blot analysis (a) and were expressed as the fold changes over the control ((b), (c), and (d)). Cont, vehicle treatment; TFCC, TFCC treatment; DOX, DOX treatment; TFCC + DOX, TFCC and DOX cotreatment. The results are represented as the mean ± SE. ^#^ *P* < 0.05 relative to the control group, and ^*^ *P* < 0.05 relative to the DOX group.

MAPK plays a key role in DOX-induced apoptosis [30]. To determine the involvement of the MAPK signaling pathway, the phosphorylation of ERK1/2, p38, and JNK kinases was examined in H9c2 cells of the control, TFCC, DOX, and TFCC + DOX groups. As shown in Figures 9(a) to 9(d), DOX induced a significant increase in the phosphorylation of ERK1/2, JNK, and p38 (after 24 h incubation) and was neutralized by TFCC pretreatment. However, TFCC alone had no influence on MAPK activation (Figures 9(a) to 9(d)).

### 3.10. Involvement of PI3K/Akt in DOX-Induced Apoptosis

**Figure 10:**
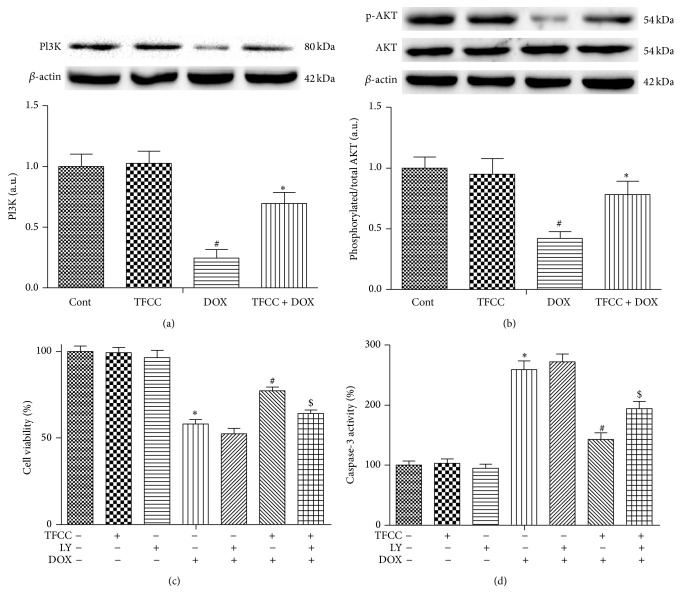
Effects of DOX and TFCC on PI3K/AKT signaling pathway activation* in vitro*. H9c2 cells were treated with vehicle or DOX (1 *μ*M) with or without TFCC (25 *μ*g/mL for 4 h prior to DOX exposure) for 24 h. The PI3K and AKT protein levels were measured using Western blot analysis and were expressed as the fold changes over the control ((a) and (b)). H9c2 cells were pretreated with 10 mM LY294002 for 30 min and then treated with DOX (1 *μ*M) with or without TFCC (25 *μ*g/mL for 4 h prior to DOX exposure) for 24 h. Cell viability and caspase-3 activity were measured ((c) and (d)). Cont, vehicle treatment; TFCC, TFCC treatment; DOX, DOX treatment; TFCC + DOX, TFCC and DOX cotreatment. The results are represented as the mean ± SE. ^#^ *P* < 0.05 relative to the control group, ^*^ *P* < 0.05 relative to the DOX group, and ^$^ *P* < 0.05 relative to the TFCC + DOX group.

We next investigated whether PI3K/AKT signaling pathway was involved in the protective effect of TFCC on DOX-induced cardiotoxicity. Results showed that DOX significantly decreased AKT phosphorylation and PI3K protein levels (Figures 10(a) and 10(b)), whereas TFCC pretreatment significantly increased the phosphorylation of these proteins. Next, we evaluated the effects of TFCC and DOX on cell viability and caspase-3 activation in cardiomyocytes with or without LY294002 (specific inhibitor of PI3K/AKT). The results showed that LY294002 pretreatment significantly inhibited the protective effect of TFCC against DOX-induced death and apoptosis in H9c2 cells. However, LY294002 alone had no influence on cell viability and caspase-3 activation (Figures 10(c) and 10(d)). These results indicated that the PI3K/AKT activation may participate in the cardioprotective effects of TFCC.

### 3.11. TFCC Had No Effect on DOX Antitumor Activity

**Figure 11:**
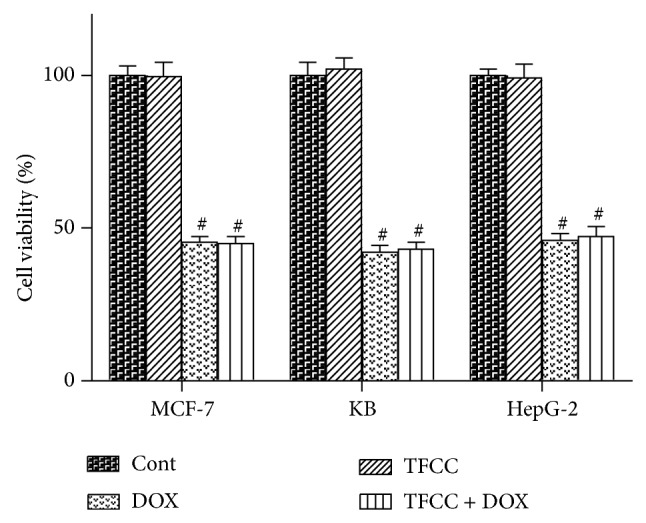
Effect of TFCC on the antitumor ability of doxorubicin. A series of cancer cell lines, including KB, HepG2, and MCF-7 cells, were treated with DOX (1 *μ*M) with or without TFCC (25 *μ*g/mL for 4 h prior to DOX exposure) for 24 h. Cell viability was determined using MTT assay (expressed as the percentage of control in each cell line). Cont, vehicle treatment; TFCC, TFCC treatment; DOX, DOX treatment; TFCC + DOX, TFCC and DOX cotreatment. Results are represented as the mean ± SE. ^#^ *P* < 0.05 relative to the control group, and ^*^ *P* < 0.05 relative to the DOX group.

We subsequently evaluated the effect of TFCC on antitumor ability of DOX. As shown in Figure 11, DOX exhibited significant inhibitory effects on the viability of different cancer cell lines, including MCF-7, KB, and HepG2 cells. Cotreatment with TFCC had no effect on DOX-induced growth delay of these cells. TFCC alone also did not affect the growth of these cells. These results suggested that TFCC had no influence on DOX antitumor effect.

## 4. Discussion

First, our study explored the protective effects of TFCC on DOX-induced myocardial injury. Further study illustrated that TFCC counteract against oxidative stress induced by DOX partially via the mitochondrion-dependent and the p53-mediated apoptotic signaling. Additionally, TFCC also activated the PI3K/AKT signaling pathways which benefit cell survival. These results suggested that TFCC could be a possible antidote to DOX-induced cardiotoxicity in clinical practice.

DOX, used in cancer patients, might induce cardiomyopathy, which can lead to heart failure [2, 31, 32]. The results demonstrated that DOX administration increased the serum levels of LDH, AST, and CK. The increase of these enzymes can cause heart failure. Besides, it could also increase cardiac index and induce histological damage in the heart. However, TFCC pretreatment can reverse this pathophysiological condition. Experiments* in vitro* also proved that pretreatment with TFCC can effectively increase cell viability and suppress DOX-induced LDH release in H9c2 cells. ROS overproduction and subsequent oxidative stress serve important functions in the initiation and progression of DOX-induced myocardial dysfunction [10]. The toxic effect of DOX on cardiomyocytes is largely attributed to its inherent chemical structure, which induces the generation of free radicals to provoke cell apoptosis [6]. In accordance with the previous study, we also proved that treatment with DOX could increase the production of oxidative free radicals and depressed expression of antioxidant enzymes in heart tissue and cardiomyocytes, indicating that DOX induced oxidative stress on myocardial cells through both evoking free radical generation and impeding intrinsic protective antioxidant capacity [33–35]. Administration of the antioxidant agent may prevent heart dysfunction in doxorubicin-treated rats [36]. Numerous studies investigated the cardioprotective properties of plant-derived polyphenolic nonsteroidal compounds, including flavonoids. It has been demonstrated that pretreatment with these agents elicits strong antioxidant activities [31, 37, 38]. In the study, TFCC could modulate the cardiomyocytes redox state by modulating the activity of enzymes involved in ROS metabolism and then effectively reduced DOX-induced alterations.

The mitochondrion is crucial in maintaining normal cell function, which is an important site of intracellular injury in cardiomyocytes after DOX exposure. Mitochondrion-dependent apoptosis in cardiomyocytes is the major factor in DOX-induced cardiotoxicity [27, 39]. We observed that DOX induced apoptosis (confirmed by DNA fragmentation analyses), upregulated proapoptotic (Bax) and downregulated antiapoptotic (Bcl-2) proteins, reduced mitochondrial membrane potential, and increased cytochrome c release into the cytosol and the cleavage of caspase-3 in cardiomyocytes. Additionally, DOX significantly increased intracellular Ca^2+^. Previous study demonstrated that cytoplasmic Ca^2+^ level is elevated after ROS accumulation, and increased Ca^2+^ influx in the mitochondria leads to mitochondrial transmembrane potential [40]. The damage of the mitochondrial transmembrane potential resulted in the release of cytochrome c from the intermembrane space to the cytoplasm, where cytochrome c induces caspase activation and provokes cell apoptosis [41]. Mitochondrion-mediated apoptosis can be modulated by the proteins of Bcl-2 family [30]. Bax translocates to the outer mitochondrial membrane and influences the permeability of the outer mitochondrial membrane in response to enhanced oxidative stress, which ultimately induces cytochrome c release from the mitochondria into the cytosol. However, Bcl-2 can stabilize the mitochondrial transmembrane potential, preserve mitochondrial integrity, and inhibit cytochrome c release [42, 43]. Thus, DOX could activate the mitochondrial-mediated apoptotic signal transduction pathways in cardiomyocytes. However, TFCC could antagonize all of these DOX-mediated proapoptotic events via inhibiting the production of ROS. Studies have shown that DOX induced apoptosis of cardiomyocytes via increasing the expression of p53 tumor suppressor protein, which activates the intrinsic apoptosis pathway [8, 44]. In the present study, we also observed that p53 expression increased in both hearts and cardiomyocytes exposed to DOX. TFCC reduced this activation of p53.

MAPK signaling pathways serve an important function in cell proliferation, differentiation, and death, which can be modulated by ROS-dependent redox cycling [45–47]. A recent study has revealed that DOX treatment increased phosphorylation of ERK1/2 persistently during the early stages of apoptosis in cardiomyocytes, whereas it had no effects on p-JNK and p-p38. However, another report has shown that p38 plays a key role in the regulation of cell apoptosis [48]. The difference in MAPK regulation may partially depend on the doses of DOX and the experimental conditions used. In the present study, TFCC pretreatment neutralized DOX-induced phosphorylation of ERK1/2, p38, and JNK, suggesting that TFCC, at least partially, prevented DOX-induced apoptosis by inhibiting the MAPK (ERK, JNK, and p38-MAPK) signaling pathway.

PI3K/Akt signaling pathway is an important cell survival signal [49]. Previous study suggested that AKT activation could prevent DOX-induced cardiomyocytes apoptosis and improve contractile function of heart [50, 51]. In this study, DOX decreased the levels of PI3K and p-AKT in cardiomyocytes compared with the control group, attenuated by pretreatment with TFCC. However, administration of the PI3K/AKT specific inhibitor blocked the protective effects of TFCC on DOX-induced cardiomyocytes apoptosis. These results suggest that TFCC protected against DOX-induced cardiotoxicity through PI3K/AKT signaling pathway activation.

At last, we discussed the effect of TFCC on the antitumor effect of DOX. A series of cancer cell lines (i.e., KB, HepG2, and MCF-7 cells) were used in the experiments. The results of* in vitro* studies have demonstrated that TFCC pretreatment has no influence on the anticancer effects of DOX. TFCC have different effects on H9c2 and tumor cells. The possible interpretation for this phenomenon may be that the effect occurs in a cell-dependent manner. However, the mechanism needs further investigation.

Our experiments demonstrated that TFCC prevented DOX-induced cardiomyocytes apoptosis* in vivo* and* in vitro*. The protective effects are partially attributed to p53-mediated inhibition, mitochondrion-dependent intrinsic apoptotic signaling, and MAPK signaling pathway involvement. TFCC can also increase PI3K/AKT phosphorylation, which promotes cell survival. Therefore, the results of the present study elucidate the fact that TFCC may have implications in the long-term clinical use of DOX.

## Supplementary Material

DOX-induced cardiomyocytes apoptosis are partially attributed to ROS production, which activate p53 and MAPK signaling pathway and ultimately trigger mitochondrion-dependent intrinsic apoptotic signaling. However, TFCC can significantly inhibit DOX-induced ROS production and DNA damage, and then inhibit p53 and MAPK-mediated, and mitochondrion-dependent intrinsic apoptotic signaling. TFCC can also increase PI3K/AKT phosphorylation, which promotes cell survival.

## Figures and Tables

**Table 1:** The MS^*n*^ data and proposed compounds of 28 peaks in total flavonoids from *Clinopodium chinense* (Benth.) O. Ktze (TFCC).

Number	*t*_*R*_ (min)	Measured mass (Da)	Calculated mass (Da)	Fragments in MS	Proposed compounds
1	5.01	593.15	593.15	447.09, 285.04	Kaempferol-3-*O*-rhamnopyranoside-7-*O*-glucopyranoside (*or* kaempferol-3-*O*-rhamnopyranoside-7-*O*-glucopyranoside isomer)

2	5.57	593.15	593.15	447.10, 285.05	Kaempferol-3-*O*-rhamnopyranoside-7-*O*-glucopyranoside (*or* kaempferol-3-*O*-rhamnopyranoside-7-*O*-glucopyranoside isomer)

3	6.00	595.17	595.17	433.16, 287.06	Eriocitrin

4	6.45	595.17	595.17	449.11, 287.06	Neoeriocitrin

5	6.64	463.09	463.09	417.21, 371.21, 287.06	Tetrahydroxyflavanone-7-O-glucuronide

6	6.92	593.15	593.15	285.04	Luteolin-7-O-rutinoside (*or* luteolin-7-O-neohesperidoside)

7	6.97	593.15	593.15	285.04	Luteolin-7-O-rutinoside (*or* luteolin-7-O-neohesperidoside)

8	7.41	579.17	579.17	271.06	Narirutin (*or* naringin)

9	8.13	579.17	579.17	271.06	Narirutin (*or* naringin)

10	8.42	609.18	609.18	579.16, 433.10, 301.07, 271.06	Hesperidin

11	8.52	609.18	609.18	433.11, 301.07, 271.06	Neohesperidin

12	8.63	577.16	577.16	269.05	Apigenin-7-O-rutinoside (*or* apigenin-7-O-neohesperidoside)

13	8.70	577.16	577.16	269.05	Pigenin-7-O-rutinoside (*or* apigenin-7-O-neohesperidoside)

14	8.81	447.09	447.09	271.06	Naringenin-7-O-glucuronide (*or* its isomer)

15	8.93	447.09	447.09	271.06	Naringenin-7-O-glucuronide (*or* its isomer)

16	9.33	577.16	577.16	269.05	Apigenin-7-O-rutinoside isomer

17	10.01	607.17	607.17	299.06	Diosmin/chrysoeriol-7-O-rutinoside

18	10.24	445.08	445.08	269.04	Apigenin-7-O-glucuronide

19	11.16	461.07	461.07	285.04	Luteolin-7-O-glucuronide

20	13.87	593.19	593.19	285.08	Didymin (*or* poncirin)

21	14.17	593.19	593.19	285.08	Didymin (*or* poncirin)

22	14.44	591.17	591.17	283.06	Linarin (*or* its isomer)

23	14.51	591.17	591.17	283.06	Linarin (*or* its isomer)

24	15.10	447.13	447.13	285.08	Isosakuranetin-7-O-glucopyranoside

25	15.87	491.10	491.10	327.22	Isosalvianolic acid C

26	16.34	271.06	271.06		Naringenin

27	16.78	269.04	269.04		Apigenin

28	19.55	285.08	285.08		Isosakuranetin

## References

[1] Gilleron M., Marechal X., Montaigne D., Franczak J., Neviere R., Lancel S. (2009). NADPH oxidases participate to doxorubicin-induced cardiac myocyte apoptosis. *Biochemical and Biophysical Research Communications*.

[2] Yang J., Maity B., Huang J. (2013). Superparamagnetic submicro-megranates: Fe_3_O_4_ nanoparticles coated with highly cross-linked organic/inorganic hybrids. *Cancer Research*.

[3] Sawyer D. B., Fukazawa R., Arstall M. A., Kelly R. A. (1999). Daunorubicin-induced apoptosis in rat cardiac myocytes is inhibited by dexrazoxane. *Circulation Research*.

[4] Berthiaume J. M., Wallace K. B. (2007). Adriamycin-induced oxidative mitochondrial cardiotoxicity. *Cell Biology and Toxicology*.

[5] Ferreira A. L. A., Yeum K.-J., Matsubara L. S. (2007). Doxorubicin as an antioxidant: maintenance of myocardial levels of lycopene under doxorubicin treatment. *Free Radical Biology and Medicine*.

[6] Takemura G., Fujiwara H. (2007). Doxorubicin-induced cardiomyopathy from the cardiotoxic mechanisms to management. *Progress in Cardiovascular Diseases*.

[7] Xiao J., Sun G. B., Sun B. (2012). Kaempferol protects against doxorubicin-induced cardiotoxicity in vivo and in vitro. *Toxicology*.

[8] Liu T.-J., Yeh Y.-C., Ting C.-T. (2008). *Ginkgo biloba* extract 761 reduces doxorubicin-induced apoptotic damage in rat hearts and neonatal cardiomyocytes. *Cardiovascular Research*.

[9] Wang Y., Su B., Sah V. P., Brown J. H., Han J., Chien K. R. (1998). Cardiac hypertrophy induced by mitogen-activated protein kinase kinase 7, a specific activator for c-Jun NH_2_-terminal kinase in ventricular muscle cells. *The Journal of Biological Chemistry*.

[10] Ghosh J., Das J., Manna P., Sil P. C. (2011). The protective role of arjunolic acid against doxorubicin induced intracellular ROS dependent JNK-p38 and p53-mediated cardiac apoptosis. *Biomaterials*.

[11] Uchiyama T., Engelman R. M., Maulik N., Das D. K. (2004). Role of Akt signaling in mitochondrial survival pathway triggered by hypoxic preconditioning. *Circulation*.

[12] Kitamura Y., Koide M., Akakabe Y. (2014). Manipulation of cardiac phosphatidylinositol 3-kinase (PI3K)/Akt signaling by apoptosis regulator through modulating IAP expression (ARIA) regulates cardiomyocyte death during doxorubicin-induced cardiomyopathy. *The Journal of Biological Chemistry*.

[13] Li G. (1989). Hemostatic and anti-inflammatory effect of *Clinopodium polycephalum*. *Journal of Biology*.

[14] Li G. (1993). Inhibitative effect of total saponins of Clinopodium poly-cephalum on immune function. *Chinese Traditional and Herbal Drugs*.

[15] Tian D. N., Wu F. H., Ma S. C., Li D., Dai Y. (2008). Studies on anti-hyperglycemic effect and its mechanism of *Clinopodium chinense*. *China Journal of Chinese Materia Medica*.

[16] Chen K., Wu F. H., Yan H. S., Qu W., Liang J. Y. (2012). Advances in studies on chemical constituents in Clinopodium and their pharmacological activities. *Strait Pharmaceutical Journal*.

[17] Dzhambazov B., Daskalova S., Monteva A., Popov N. (2002). *In vitro* screening for antitumour activity of *Clinopodium vulgare* L. (Lamiaceae) extracts. *Biological and Pharmaceutical Bulletin*.

[18] Zhong M., Sun G., Zhang X., Xu X., Yu S. (2012). A new prenylated naphthoquinoid from the aerial parts of *Clinopodium chinense* (Benth.) O. Kuntze. *Molecules*.

[19] Li G. X. (1993). Cardiovascular system action of total flavones of *Clinopodium polycephalum*. *Journal of Anhui University (Natural Science Edition)*.

[20] Zhu H. L., Meng Z. Q., Ding G., Xiao W. (2013). Progress in research of clinopodii herba. *World Science and Technology/Modernization of Traditional Chinese Medicine and Materia Medica*.

[21] Sun X., Sun G. B., Wang M., Xiao J., Sun X.-B. (2011). Protective effects of cynaroside against H_2_O_2_-induced apoptosis in H9c2 cardiomyoblasts. *Journal of Cellular Biochemistry*.

[22] Wang M., Sun G.-B., Sun X. (2013). Cardioprotective effect of salvianolic acid B against arsenic trioxide-induced injury in cardiac H9c2 cells via the PI3K/Akt signal pathway. *Toxicology Letters*.

[23] Ma C., Gao W., Gao Y., Man S., Huang L., Liu C. (2011). Identification of chemical constituents in extracts and rat plasma from Fructus Aurantii by UPLC-PDA-Q-TOF/MS. *Phytochemical Analysis*.

[24] Shi P., He Q., Song Y., Qu H., Cheng Y. (2007). Characterization and identification of isomeric flavonoid O-diglycosides from genus Citrus in negative electrospray ionization by ion trap mass spectrometry and time-of-flight mass spectrometry. *Analytica Chimica Acta*.

[25] Li X., Lin J., Han W. (2012). Antioxidant ability and mechanism of rhizoma *Atractylodes macrocephala*. *Molecules*.

[26] Cao J., Xia X., Chen X., Xiao J., Wang Q. (2013). Characterization of flavonoids from *Dryopteris erythrosora* and evaluation of their antioxidant, anticancer and acetylcholinesterase inhibition activities. *Food and Chemical Toxicology*.

[27] Green P. S., Leeuwenburgh C. (2002). Mitochondrial dysfunction is an early indicator of doxorubicin-induced apoptosis. *Biochimica et Biophysica Acta—Molecular Basis of Disease*.

[28] Ueno M., Kakinuma Y., Yuhki K.-I. (2006). Doxorubicin induces apoptosis by activation of caspase-3 in cultured cardiomyocytes in vitro and rat cardiac ventricles in vivo. *Journal of Pharmacological Sciences*.

[29] Yeh Y.-C., Lai H.-C., Ting C.-T. (2007). Protection by doxycycline against doxorubicin-induced oxidative stress and apoptosis in mouse testes. *Biochemical Pharmacology*.

[30] Das J., Ghosh J., Manna P., Sil P. C. (2011). Taurine suppresses doxorubicin-triggered oxidative stress and cardiac apoptosis in rat via up-regulation of PI3-K/Akt and inhibition of p53, p38-JNK. *Biochemical Pharmacology*.

[31] van Acker F. A. A., van Acker S. A. B. E., Kramer K., Haenen G. R. M. M., Bast A., van Der Vijgh W. J. F. (2000). 7-Monohydroxyethylrutoside protects against chronic doxorubicin-induced cardiotoxicity when administered only once per week. *Clinical Cancer Research*.

[32] Wold L. E., Aberle N. S., Ren J. (2005). Doxorubicin induces cardiomyocyte dysfunction via a p38 MAP kinase-dependent oxidative stress mechanism. *Cancer Detection and Prevention*.

[33] Chaiswing L., Cole M. P., St. Clair D. K., Ittarat W., Szweda L. I., Oberley T. D. (2004). Oxidative damage precedes nitrative damage in adriamycin-induced cardiac mitochondrial injury. *Toxicologic Pathology*.

[34] Aldieri E., Bergandi L., Riganti C., Costamagna C., Bosia A., Ghigo D. (2002). Doxorubicin induces an increase of nitric oxide synthesis in rat cardiac cells that is inhibited by iron supplementation. *Toxicology and Applied Pharmacology*.

[35] Han X., Pan J., Ren D., Cheng Y., Fan P., Lou H. (2008). Naringenin-7-O-glucoside protects against doxorubicin-induced toxicity in H9c2 cardiomyocytes by induction of endogenous antioxidant enzymes. *Food and Chemical Toxicology*.

[36] Brilhante Wolle C. F., de Aguiar Zollmann L., Etges A., Vitalis G. S., Leite C. E., Campos M. M. (2012). Effects of the antioxidant agent tempol on periapical lesions in rats with doxorubicin-induced cardiomyopathy. *Journal of Endodontics*.

[37] Bast A., Haenen G. R. M. M., Bruynzeel A. M. E., van der Vijgh W. J. F. (2007). Protection by flavonoids against anthracycline cardiotoxicity: from chemistry to clinical trials. *Cardiovascular Toxicology*.

[38] Bast A., Kaiserová H., den Hartog G. J. M., Haenen G. R. M. M., van der Vijgh W. J. F. (2007). Protectors against doxorubicin-induced cardiotoxicity: flavonoids. *Cell Biology and Toxicology*.

[39] Nithipongvanitch R., Ittarat W., Cole M. P., Tangpong J., Clair D. K. S., Oberley T. D. (2007). Mitochondrial and nuclear p53 localization in cardiomyocytes: redox modulation by doxorubicin (Adriamycin)?. *Antioxidants and Redox Signaling*.

[40] Gharanei M., Hussain A., Janneh O., Maddock H. L. (2013). Doxorubicin induced myocardial injury is exacerbated following ischaemic stress via opening of the mitochondrial permeability transition pore. *Toxicology and Applied Pharmacology*.

[41] Kluza J., Marchetti P., Gallego M.-A. (2004). Mitochondrial proliferation during apoptosis induced by anticancer agents: effects of doxorubicin and mitoxantrone on cancer and cardiac cells. *Oncogene*.

[42] Hsu L.-J., Hong Q., Schultz L. (2008). Zfra is an inhibitor of Bcl-2 expression and cytochrome c release from the mitochondria. *Cellular Signalling*.

[43] Korytowski W., Basova L. V., Pilat A., Kernstock R. M., Girotti A. W. (2011). Permeabilization of the mitochondrial outer membrane by bax/truncated Bid (tBid) proteins as sensitized by cardiolipin hydroperoxide translocation: mechanistic implications for the intrinsic pathway of oxidative apoptosis. *The Journal of Biological Chemistry*.

[44] Liu X. W., Chua C. C., Gao J. P. (2004). Pifithrin-*α* protects against doxorubicin-induced apoptosis and acute cardiotoxicity in mice. *American Journal of Physiology—Heart and Circulatory Physiology*.

[45] Wagner E. F., Nebreda Á. R. (2009). Signal integration by JNK and p38 MAPK pathways in cancer development. *Nature Reviews Cancer*.

[46] Choi T. G., Lee J., Ha J., Kim S. S. (2011). Apoptosis signal-regulating kinase 1 is an intracellular inducer of p38 MAPK-mediated myogenic signalling in cardiac myoblasts. *Biochimica et Biophysica Acta—Molecular Cell Research*.

[47] Bhattacharyya S., Ghosh J., Sil P. C. (2012). Iron induces hepatocytes death via MAPK activation and mitochondria-dependent apoptotic pathway: beneficial role of glycine. *Free Radical Research*.

[48] Thandavarayan R. A., Watanabe K., Sari F. R. (2010). Modulation of doxorubicin-induced cardiac dysfunction in dominant-negative p38*α* mitogen-activated protein kinase mice. *Free Radical Biology and Medicine*.

[49] Kennedy S. G., Wagner A. J., Conzen S. D. (1997). The PI 3-kinase/Akt signaling pathway delivers an anti-apoptotic signal. *Genes and Development*.

[50] Shiojima I., Schiekofer S., Schneider J. G. (2012). Short-term Akt activation in cardiac muscle cells improves contractile function in failing hearts. *American Journal of Pathology*.

[51] Hong H.-J., Liu J.-C., Chen P.-Y., Chen J.-J., Chan P., Cheng T.-H. (2012). Tanshinone IIA prevents doxorubicin-induced cardiomyocyte apoptosis through Akt-dependent pathway. *International Journal of Cardiology*.

