# Supplementary figures and images for: Total Flavonoids from Clinopodium chinense (Benth.) O. Ktze Protect against Doxorubicin-Induced Cardiotoxicity In Vitro and In Vivo
Source: Evid Based Complement Alternat Med. 2015 Feb 16;2015:472565. doi: 10.1155/2015/472565 (PMC4346128; doi:10.1155/2015/472565)

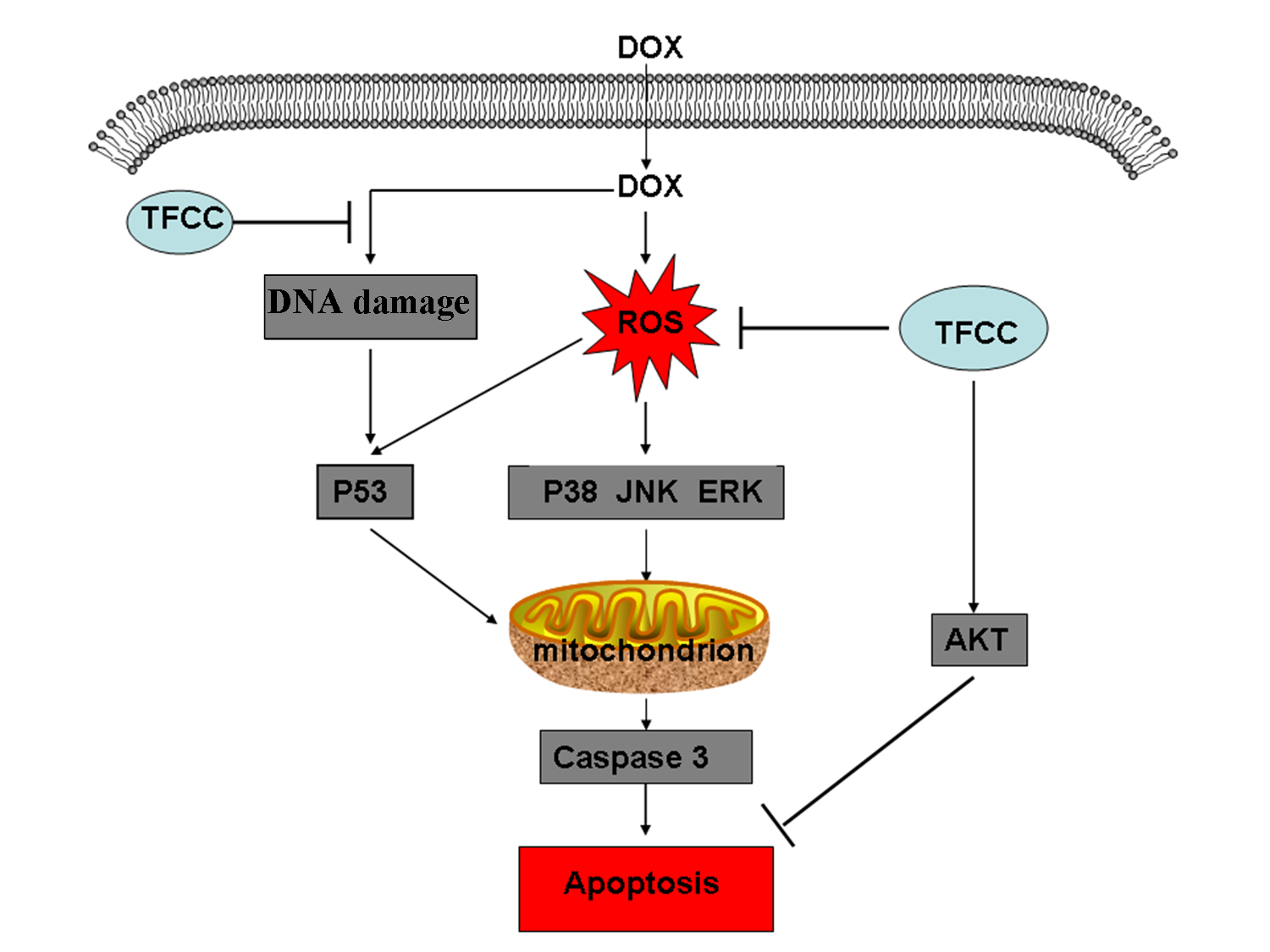

Supplement: Supplementary file 1 — DOX-induced cardiomyocytes apoptosis are partially attributed to ROS production, which activate p53 and MAPK signaling pathway and ultimately trigger mitochondrion-dependent intrinsic apoptotic signaling. However, TFCC can significantly inhibit DOX-induced ROS production and DNA damage, and then inhibit p53 and MAPK-mediated, and mitochondrion-dependent intrinsic apoptotic signaling. TFCC can also increase PI3K/AKT phosphorylation, which promotes cell survival. [file 472565.f1.tif]
